# Cytosolic RNA:DNA Duplexes Generated by Endogenous Reverse Transcriptase Activity as Autonomous Inducers of Skin Inflammation in Psoriasis

**DOI:** 10.1371/journal.pone.0169879

**Published:** 2017-01-17

**Authors:** Jean-Pierre Molès, Anthony Griez, Jean-Jacques Guilhou, Céline Girard, Nicolas Nagot, Philippe Van de Perre, Pierre Dujols

**Affiliations:** 1 Inserm UMR 1058, Montpellier, France; 2 Etablissement Français du Sang, Montpellier, France; 3 University of Montpellier, Montpellier, France; 4 CHU of Montpellier, Montpellier, France; Plymouth University, UNITED KINGDOM

## Abstract

Psoriasis is a chronic skin disease of unknown ætiology. Recent studies suggested that a large amount of cytosolic DNA (cyDNA) in keratinocytes is breaking keratinocytes DNA tolerance and promotes self-sustained inflammation in the psoriatic lesion. We investigated the origin of this cyDNA. We show that, amongst all the possible DNA structures, the cyDNA could be present as RNA:DNA duplexes in keratinocytes. We further show that endogenous reverse transcriptase activities generate such duplexes and consequently activate the production of Th1-inflammatory cytokines. These observations open a new research avenue related to endogenous retroelements for the aetiology of psoriasis and probably of other human chronic inflammatory diseases.

## Introduction

Psoriasis is a chronic inflammatory skin disease mediated by dendritic and T-cells and involving a self-sustained cross-talk between the innate and the adaptive immune systems. It results in intense proliferation of keratinocytes and their impaired differentiation program. This cross-talk involves the production of various Th1, Th17 and Th22 cytokines depending on the stage of the disease, *i*.*e*. initiation phase or maintenance phase [1–3]. The importance of these events is supported by recent studies associating psoriasis susceptibility to genes involved in innate and adaptive immunity as well as skin barrier functions [4,5].

Several environmental factors have been reported to trigger and/or exacerbate inflammation in psoriatic skin including bacterial infections, such as rhino-pharyngeal β-hemolytic streptococcus prior to the onset of psoriasis of the child [6], or viral infections, such as human immunodeficiency virus (HIV) infection associated with the initiation or worsening of psoriasis [7]. So far, the prime mechanism by which psoriasis is initiated remains to be identified.

Recently, several reports suggested that a large amount of cytosolic DNA (cyDNA) may promote inflammation in the psoriatic lesion [8–10] by breaking keratinocyte cyDNA tolerance, activating Toll-like receptor (TLR) and AIM2 pathways [11,12], then triggering and sustaining the inflammatory loop [13,14]. These events were partly confirmed in various experimental models by means of TLR inhibitors. TLR-8 and TLR-9 antagonists, tested in an IL23 psoriasis mouse model, inhibited the Th1 and Th17 responses [15,16], and were active against psoriasis in a proof-of-concept trial [17]. These data suggest that upstream to the self-sustained inflammatory loop, cyDNA, amongst other danger-associated molecular pattern molecules, can be responsible for the initiation of psoriatic lesions.

Various hypotheses regarding the source of these cyDNAs have been developed. It may originate from the extra-cellular compartment, as a result of fungal or bacterial infection, or as a result of cell damage. LL37, an antimicrobial peptide overexpressed in psoriatic lesions, is able to disrupt bacterial membranes leading to free circulating DNA. Moreover, LL37 can complex with extracellular host nucleic acids *in vitro*, allowing their entry into the intracellular compartment containing TLR7/8/9 or other cyDNA sensors [14]. Alternatively, cyDNA may arise directly from the intracellular compartment, resulting either from a viral infection or from extensive-DNA replication generating ssDNA or dsDNA byproducts. However, *in vivo*, ssDNA or dsDNA in the cytosol have not been firmly demonstrated in keratinocytes of psoriatic lesions.

Recently, TLR9, known to interact with DNA structures, has been shown *in vitro* to interact with cytosolic RNA:DNA duplexes and to trigger cytokine expression in bone marrow derived Flt3-L dendritic cells [18]. We have previously demonstrated that reverse transcriptase (RT) activities, which produce such RNA:DNA intermediates are increased in psoriatic lesions compared to non lesional skin [19]. In order to elucidate an endogenous origin of the cyDNA, we therefore investigated whether such duplexes are present in the lesion.

Here, we provide the evidence that the overexpression of RNA:DNA duplexes is generated by endogenous reverse transcriptases in psoriatic lesions.

## Materials and Methods

### Tissue sample collection and cell culture

Skin biopsies were collected at the department of Dermatology, University Teaching Hospital, Montpellier, France, with the approval of the local ethic committee (Comité de protection des Personnes Sud Méditerranée IV) and the written informed consent of all participants. Biopsies from lesional psoriatic skin (n = 14) were collected at the florid margin of an active lesion, while matched non lesional skin (n = 12) samples consisted of normal skin with no history of previous lesion and located at least 5 cm away from any lesions. One additional sample referred as nonlesional skin was collected in a healed, formerly active lesion. The patients did not receive any systemic treatment for at least one month nor any topical treatment for at least one week. Normal skins (n = 12) from healthy persons were collected at the Saint Jean’s Clinic, plastic surgery department, Montpellier, France. Biopsies were embedded into OCT compound (Miles, Elkhart, IN, U.S.A.), immediately snap-frozen into liquid nitrogen and stored at -80°C until use.

Human keratinocytes were isolated from foreskin (Saint Jean’s Clinic) according to the Rheinwald & Green method [20] and subsequently cultured in K-SFM medium (Life Technologies, Saint Aubin, France). Desoxiiodouridine (200 μg/ml final), azacytidine (1.25 μg/ml final), azidothymidine were purchased from Sigma-Aldrich (Saint Quentin-Fallavier, France). GP+E-86 cell line was cultured in 10% donor calf serum in DMEM supplemented with penicillin/streptomycin (10,000 IU/ml/10,000 μg/ml; Life technologies, Saint Aubin, France).

### Immunohistochemistry

Six micrometers skin sections were fixed with 3.7% formaldehyde/PBS for 10 min. and incubated with a saturating solution (0.1% gelatin in PBS) for 2 hours in a humidified chamber. Sections were exposed overnight at 4°C to the following primary antibodies: anti-DNA:RNA hybrid antibody (clone S9.6, Kerafast Inc., Boston MA), anti-DNA:RNA hybrid antibody (clone D5H6, Covalab, Villeurbanne, France). Slides were further processed for immunofluorescence staining using Alexafluor 488-conjugated goat anti-species antibody (Molecular Probes^™^, Eugene, OR). Negative controls consisted of the same procedure but with the omission of the primary antibody. Sections were then examined with a Nikon TE300 microscope (Japan) equipped with digital camera Nikon DMX1200 (Japan). Immunohistochemistry on cell culture was done as described above except that the fixation step was performed with 3.7% formaldehyde/0.1% Triton X-100/PBS.

### Detection of In situ Reverse Transcriptase Activity (DIRTA)

Cryosections of OCT-embedded tissues were obtained using a cryomicrotome (Leica, Germany) and kept at -20°C until used. Immediately after thawing, a mixture of incubation buffer (10 μl) and reaction buffer (40 μl) was applied on top of the section and incubated at 37°C for 1 hour in a humid chamber. Incubation buffer consists of sucrose 250 mM, NaCl 75 mM, spermidine 0.5 mM, spermine 0.15 mM and BSA 3%. The reaction buffer consists of KHEPES 40 mM pH 7.8, MgCl_2_ 7 mM or MnCl_2_ 100 mM, a nucleotid mix (ATP 30mM, dATP, dGTP and dTTP 1 mM each; Promega, Lyon, France), biotinylated dCTP 10 μM (Invitrogen^™^, Saint Aubin, France), DTT 1 mM, creatine phosphate 40 mM, phosphocreatine kinase 5 μg/ml and yeast tRNA 0.1 mg/ml. Ongoing reactions were then stopped by 3 washes in PBS, fixed in 3.7% formaldehyde (10 min.) followed by cold methanol for 5 min. After inhibition of endogenous peroxidases, incorporated tagged nucleotide were next revealed by incubation with a streptavidin-HRP and by a colorometric reaction with AEC chromogen. Nuclei were counterstained with aqueous hematoxilin (DiaPath, Italy). As an alternative, the use of DIG-11-dUTP (Roche diagnostics, Penzberg, Germany) as tagged nucleotide was conducted with a revelation step done with an anti-DIG antibody (Boehringer, Mannhein, France) and an Alexafluor 488-conjugated goat anti-species antibody (Molecular Probes^™^, Eugene, OR). The packaging cell line GP+E-86 was used as positive control, experiments with the omission of biotinylated dCTP was the negative control. Most of the reagents were purchased from Sigma-Aldrich (Saint Quentin Fallavier, France) except when specified.

### DNA copy number

Cells were grown up to 80% confluence and then treated with demethylating agents, namely azacytidine and/or iododesoxyuridine for 24, 48 and 72 hours. Cells were next harvested for DNA extraction and cell culture supernatants collected for cytokine detection. DNA extraction was performed using Qiagen mini DNA extract kit according to the manufacturer’s instructions and quantified by a Biophotometer plus (Eppendorf, Hamburg, Germany). Relative copy numbers was quantified by real time PCR on HERV-K and LINE-1 targets. Briefly, real-time PCR was performed using the LightCycler^®^480 probes master on 20 ng of total DNA with the LightCycler^®^480 II (Roche Diagnostics, Mannheim, Germany). Sample were run in triplicate with the following primers LINE-1 forward 5’-CAAACACCGCATATTCTCACTCA-3’, reverse 5’-CTTCCTGTGTCCATGTGATCTCA-3’, probe 5’-(FAM)AGGTGGGAATTGAC(TAMRA)-3’; primers HERV-K forward 5’-ATTGGCAACACCGTATTCTGCT-3’, reverse 5’-CAGTCAAAATATGGACGGATGGT-3’, probe 5’-(FAM)ACACAGGGATCCACACG(TAMRA)-3’; GAPDH forward 5’-GAAGGTGAAGGTCGCAGT-3’, reverse 5’-GAGATGGTGATGGGATTTC-3’, probe 5’-(FAM)CAAGCTTCCCGTTCTCAGCC(TAMRA)-3’. Relative quantification of each target was determined using the comparative cycle threshold (Ct) (2^-ΔΔCt^) method after correction by the efficiency of both amplifications (E-method). The ΔCt was obtained by subtracting the mean GAPDH reference Ct value from the average Ct value of each target. The average ΔCt of the untreated cells was used as the calibrator. This study showed copy number results as fold changes, calculated according to the formula 2^-ΔΔCt^, where ΔΔCt was the difference between ΔCt and the ΔCt calibrator value.

### Cytokine expression

Cell culture supernatants were centrifuged at 2,500 *g* for 2 minutes, aliquoted and stored at -80°C until further analysis. Quantitative measurement of IL-1RA, IL-6, IL-8, IL-17A, IL-33, IFN-γ, IP10 and TNF-α was assayed by enzyme-linked immunosorbent assay (Human Standard ELISA Development Kit, PeproTech Inc., Rocky Hill, CT, USA) according to the manufacturer’s instructions, and read out using a microplate reader (Multiskan FC, Thermo Scientific, Saint Herblain, France). Samples were tested in duplicate in three independent experiments, and a calibration curve was added to each run.

## Results

### RNA:DNA duplexes in psoriatic lesions

**Fig 1:**
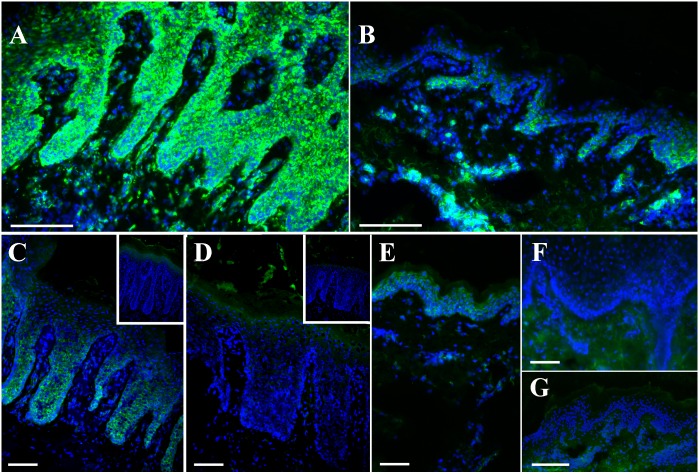
Detection of RNA:DNA duplexes in psoriatic lesions, healed psoriatic lesions and normal skin. Using indirect immunofluorescence, staining was observed in the epidermis of lesional psoriatic skin (A, MAb clone S9.6; C, MAb clone D5H6), but not DNase1 pretreated skin section (D) or in non lesional psoriatic skin (E) or in atopic dermatitis (F). Focal staining was noted in skin section from a formally lesional psoriatic skin, both in the epidermis and in the underlying residual inflammation (B). Negative control is shown in panel G. The bar represents 80 μm.

During its initial step, reverse transcription produces RNA:DNA duplexes as an intermediate product. We therefore hypothesized that, if RT activity accounts for the production of a cytosolic pool of DNA, RNA:DNA duplexes should be detected in skin lesions. By immunofluorescence using two distinct monoclonal antibodies raised against RNA:DNA duplexes, a strong cytoplasmic staining in the basal and upper layers of psoriatic epidermis was observed (Fig 1A and 1C). Positive cells in the dermis were observed with the S9.6 antibody while it was less frequent with the D5H6 antibody. However, in non lesional skin and in atopic dermatitis sections, the signal was barely detectable (Fig 1E and 1F). Staining was also detected in the rete ridges of a cleared lesional skin, as well as in the underlying inflammatory dermal cells (Fig 1B). When DNase I treatment was initiated before the incubation of the primary antibody, no staining was observed (Fig 1D).

### Localisation of the endogenous reverse transcriptase activities

**Fig 2:**
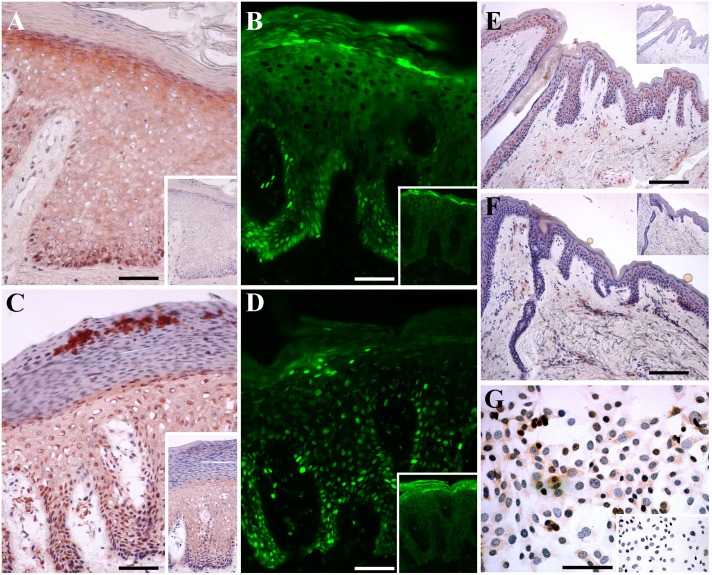
Detection of *in situ* RT activities in lesional psoriatic skins and normal skin. On-going RT reactions were visualized by the detection of a tagged nucleotide, biotinylated dCTP and revealed by immunohistochemistry (ACEFG), or DIG-11-dUTP revealed by immunofluorescence (BD). Mn^2+^-dependent RT is shown in ABE while Mg^2+^-dependent RT is shown in CDFG. AC, BD and EF were issued from the same biopsies. GP+E-86 packaging cell line was used as positive control. Negative controls consisted of the same procedure but with omission of the primary antibody. They are shown as insert of each panel.

An *in situ* detection technique for RT activity was developed based on a previously described technique for the detection of DNA-dependent DNA-polymerases [21]. In principle, salts, energy and reagents are provided to allow RNA-dependent DNA-polymerases to achieve their on-going synthesis. In the meantime, a tagged nucleotide is introduced in the reaction to lately localize the end-products. Reaction buffers were chosen amongst those used in conventional RT assays to investigate both Mg^2+^ and Mn^2+^-dependent RT activities. Stained cells were observed in lesional skin sections from psoriatic patients and from healthy controls. Both Mg^2+^ and Mn^2+^-dependent RT activities were detected but differed in localization (Fig 2). The Mn^2+^ RT activity was mainly observed in the upper layers of the psoriatic epidermis with a clear cytosolic localization. Additional cells with a strong nuclear staining were present in basal and first suprabasal layers. No staining was observed in the dermis (Fig 2A and 2B). Epidermis of the normal skin contained few faintly positive cells. On the other hand, the Mg^2+^ RT activity was only seen in the nucleus of cells localized in the proliferative layer of the epidermis, but also of neutrophils included in Munro-Saboureau abscesses (Fig 2C and 2D). Non lesional skin sections of psoriatic patients showed fainter staining, mostly nuclear in the epidermis and in rare dermal cells. The Mg^2+^ RT activity was almost undetectable in normal skin with this technique (Fig 2E and 2F). These observations were independent of the nature of the tagged nucleotide, since the use of DIG-11-dUTP (Fig 2B and 2D) confirmed the results obtained with biotinylated-dCTP (Fig 2A and 2C).

### RNA:DNA duplexes induce cytokine expression in keratinocytes

**Fig 3:**
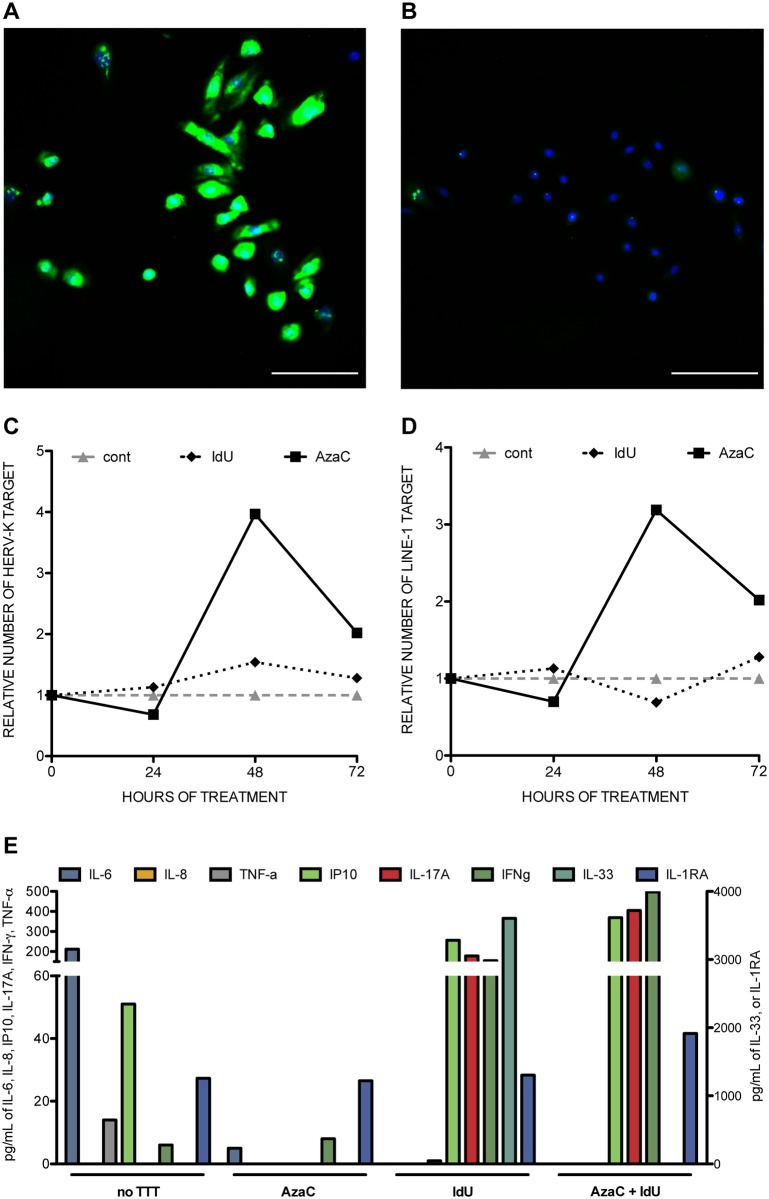
RNA:DNA duplexes and cytokine secretion. Human keratinocytes were incubated with 1.25 μg/ml of AzaC, then stained with MAb clone S9.6 and revealed by immunohistochemistry. A shows induction of RNA:DNA synthesis when compared to control experiment (B). Nuclei were counterstained with Hoescht 33258. Molecular quantification by real time PCR was next performed on two target genes, namely *HERV-K* (C) and *LINE-1* (D). Keratinocytes were treated by AzaC (1.25 μg/ml) or by IdU (200 μg/ml) and total nucleic acid were extracted. Both diagrams show the evolution of the ratio target gene to *GAPDH* over 72 hours. Cytokine concentrations were measured in the supernatant of the above treated cells at 48 hours (E).

We hypothesized that RNA:DNA duplexes can trigger cutaneous inflammation in primary cultured keratinocytes from healthy donors, as cyDNA does [8–10]. Our first challenge was to recreate an endogenous RT activity since these enzymes displaying RT activity are currently undetermined. Demethylating agent exposure to various cell lines is able to induce such RT activities [22]. Amongst them, azacytidine (AzaC) at 1,25 μg/mL and/or desoxiuridine (IdU) at 200 μg/mL, allowed the detection RNA:DNA duplexes in the cytosol of cultured keratinocytes within 48 h (Fig 3A and 3B). We next aimed at quantifying this induction by real time PCR. Genes used in this process are unknown. We selected two genes belonging to *HERV-K* and *LINE-1* families, because the laters are known to encode for RT proteins, to use this activity for their mobility and therefore their intermediate products should exist in a RNA:DNA duplex form. The genomic DNA was specifically amplified by a couple of primers that overlaps an intron-exon junction of the *GAPDH* gene, therefore unlikely to exist as a RNA:DNA duplex form. With AzaC treatment, the copy numbers of *HERV-K* and *LINE-1* genes increased by 3.9-fold and 3.2-fold, respectively, after correcting with an internal calibrator, the *GAPDH* gene, 48h after stimulation. These DNA copy numbers decreased to baseline levels 72h after stimulation (Fig 3C and 3D). The effect of the AzaC treatment on the induction of HERV-K and LINE-1 RNA:DNA duplexes was more potent than the effect of IdU treatment. Finally, cytokine secretion was assessed in the same experimental conditions. None of the treatments, alone or combined, had an effect on the level of expression of IL-8, TNF-α or IL1-RA. However, AzaC and IdU were effective to stimulate IP-10, IL-17A, IL-33 and IFN-γ secretion and were more potent when used together (Fig 3E).

## Discussion

In addition to the individual role of ssDNA and dsDNA in the early pathogenesis of psoriasis, our study investigated the presence of RNA:DNA duplexes in the cytosol of psoriatic keratinocytes and the RT activity origin of these nucleic acids, as well as their role as inductor of inflammation.

Nucleic acids of bacterial origin have been detected in the extra-cellular compartment in psoriatic lesions. These nucleic acids are supposedly released from the skin microbiota [23,24] or from translocated bacteria in the peripheral blood [25]. Bacterial RNA:DNA duplexes are naturally formed during bacterial DNA replication and transcription. Following phagocytosis/pinocytosis, these duplexes can be sensed by NLRP3, ASC or caspase1 inflammasome complexes [26] and elicit an innate immune response. However, even if infectious processes sometimes develop concomitantly with psoriasis flare exacerbation [27], the association of psoriasis exacerbation with infections has not been confirmed in large series [7]. The bacterial origin of the RNA:DNA duplexes detected in psoriasis is therefore unlikely.

RNA:DNA duplexes are also formed as replication intermediates during retrovirus life cycle, and can contribute to NLPR3 inflammasome-mediated immune response [26]. While a cutaneous antiviral activity has been recently described in psoriatic lesions [28], no viral stimulus has been demonstrated in psoriasis pathogenesis. During the early 1980s, viral particles have been repeatedly sought by electron microscopy, which produced few suggestive images [29–31]. However, no molecular confirmation ever supported these observations. The exogenous viral origin of the RNA:DNA duplexes detected in psoriasis is therefore also unlikely.

RNA:DNA duplexes exist also naturally in eukaryotic cells. They are formed in *cis* during transcription when nascent RNA hybridizes to the DNA template behind the elongating RNA polymerase, the R-loop [32]. During this process, hybrids are located only in the nucleus and not in the cytoplasm, as observed in psoriatic lesions in our experiments (Fig 1). However, a nuclear-cytoplasmic translocation of these hybrids is not excluded, and therefore not related to the classical R-loop [32].

Here, we provide evidence for a combined origin, viral and endogenous, for these RNA:DNA duplexes and biological and clinical arguments suggest a role for endogenous RT (ERT). Indeed, ERT activities were previously reported in healthy human skin and in nonlesional psoriasis skin at very low levels but at 3-fold increase in psoriatic lesions [19]. It is also noteworthy that progeny of transgenic mice containing HIV pro-viral DNA, including the RT, develop psoriasis-like skin lesions with epidermal hyperplasia, hyperkeratosis and parakeratosis [33].

Several human proteins harbor RT activities. The telomerase is a ribonucleoprotein with a RT activity that stabilizes telomere length, protects chromosomes from degradation and has an anti-apoptotic activity. Telomerase activity has been demonstrated in keratinocytes of the proliferative basal layer of the epidermis and is increased in psoriatic skin lesions [34–36].

Retrotranposons encompass about 40% of human genome and are divided into LTR (long terminal repeat) and non-LTR sequences. The latter, found in extremely high copy numbers, are divided in short interspersed elements (SINEs) and long interspersed elements (LINEs). While SINEs are non-autonomous and have no protein coding capacity, LINEs are of interest since their open reading frame-2 (ORF) encodes for RT-like proteins. Recently, hypomethylation of LINE-1 sequences has been reported in psoriatic lesions compared to normal epidermis, while no difference could be observed in the peripheral blood cells of the same patients, suggesting that LINE-1 sequences were turned on [37].

Remnants of ancient retroviruses having lost their infecting abilities, LTR sequences or human endogenous retroviruses (HERV) represent 8% of the human genome [38]. They possess regulatory regions, LTRs and ORFs, potentially able to encode for complete *gag*, *pol* or *env* proteins with their functional activities. Although the majority of them, accumulating point mutations, insertions/deletions, are largely defective in terms of functional RT proteins, others members of the HERV-K family, such as HERV-K113 and -K115, contain complete ORFs able to generate functional RT proteins [39], not involved in a classical viral cycle but rather in a yet undefined cellular function. RT encoding genes are transcriptionally active in stem cells, in undifferentiated cells and in cells with high proliferative potential while being barely detectable in most of the differentiated cells. The highest levels of expression are found in hyperproliferative pathological states such as human cancer cells and stem cells [40,41].

Once DNA is found in the cytosol as free or complexed forms, various pathways are activated in order to eliminate this abnormality. Regarding nucleic acid sensors, TLR9 can complex with RNA:DNA duplexes and induce a cytokine cascade in response [18]. In addition, up to a certain threshold, cells can tolerate cyDNA but beyond this point, the imbalance generates an immune activation [42]. This imbalance may result from an excess of production or from a decreased elimination of cyDNA. Several human diseases have been linked to a deficit in this process, such as the Acairdi-Gouttieres syndrome associated with defects in SAMHD1, ribonuclease and other regulators of cytosolic nucleic acids. It is noteworthy that unaffected relatives of patients with the Acairdi-Gouttieres syndrome are prone to various autoimmune diseases [42]. In normal epidermis, a set of endonucleases, in particular DNase1L2 and APE1, are active [43], but this enzymatic activity is altered in psoriasis [44,45]. Therefore, an imbalanced nucleic metabolism may be involved in the accumulation of cyDNA, which would require further investigations.

HIV-infected individuals are sometimes also suffering from psoriasis. Several case reports noted rapid improvement of psoriatic lesions after initiation of an antiretroviral therapy containing RT inhibitors (RTI) [7]. A therapeutic effect of zidovudine, an RTI molecule, on psoriasis was also observed in an open-labeled clinical trial of 12 HIV-uninfected volunteers [46], but it was speculated to be attributed to a direct antimitotic effect of zidovudine on epidermal proliferation. However, recent findings suggest a new mechanism since nucleoside RTI possess an intrinsic anti-inflammatory activity by inhibiting P2X7-mediated inflammasome activation [47].

A pathogenic role for RNA:DNA duplexes in triggering other inflammatory diseases than psoriasis should also be addressed. Diseases belonging to the auto-inflammatory continuum including psoriasis and Crohn’s disease [48,49] share clinical and biological characteristics and genetic susceptibility loci. The place of ERT activity with subsequent accumulation of RNA:DNA duplexes opens new pathophysiologic hypotheses. Evaluation studies of RTI treatments in amyotrophic lateral sclerosis, multiple sclerosis or Aicardi-Gouttieres syndrome (ClinicalTrials.gov Identifier: NCT02437110, NCT01767701 and NCT02363452) are on-going. Even if their rationales are not based on the RNA:DNA duplexes accumulation triggering inflammation, they extend the interest of our findings. More fundamentally, our findings reopen the question of the regulatory role of HERV in the immune homeostasis. If confirmed, the central role of HERV expression in the control of inflammation and immune response could explain why endogenous retroviruses and hosts have coevolved over millions of years.
